# Robotic Vaginal Cuff Closure During Radical Hysterectomy for Early-Stage Cervical Cancer: The Bruges Method

**DOI:** 10.7759/cureus.49149

**Published:** 2023-11-20

**Authors:** Thaïs Lesseliers, Philippe Van Trappen

**Affiliations:** 1 Gynecology/Oncology, AZ Sint-Jan Brugge AV, Bruges, BEL

**Keywords:** vaginal cuff, robot, radical hysterectomy, early-stage, cervical cancer

## Abstract

The only randomized trial (LACC trial, Laparoscopic Approach to Cervical Cancer), published in 2018, comparing the oncologic outcomes of minimally invasive and open surgery in early-stage cervical cancer, has shown inferior disease-free and overall survival for minimally invasive surgery. Subsequent large retrospective cohort studies of centers with long-standing experience in minimally invasive surgery and large nationwide cohort studies have shown that both the laparoscopic and robotic approaches have similar survival outcomes as the open surgery group in the LACC trial. Important protective measures to avoid tumor spillage in the peritoneal cavity during colpotomy were the closure of the vaginal cuff and avoiding the use of a uterine manipulator. Several methods have been described to close the vaginal cuff, mainly by a vaginal approach. Here we describe with a video a new technique of vaginal cuff closure during a robotic-assisted radical hysterectomy. During the robotic procedure, a purse string barbed suture is placed through the vaginal walls in order to close the vagina prior to colpotomy. The technique is a feasible, relatively fast, and easy-to-learn addition to the robotic radical hysterectomy procedure in early-stage cervical cancer.

## Introduction

Minimally invasive surgery for early-stage cervical cancer was introduced in the early 1990s and, since then, widely adopted worldwide [1]. Several retrospective studies have shown no difference in survival between open radical hysterectomy (OH) vs. minimally invasive radical hysterectomy (MIH) [2,3]. However, the publication of the results of the prospective randomized trial Laparoscopic Approach for Cervical Cancer (LACC) surprisingly showed inferior disease-free and overall survival for MIH compared to OH [4]. Subsequent studies confirmed these findings [5-7]. In light of these findings, the European Society of Gynaecological Oncology (ESGO) and the National Comprehensive Cancer Network (NCCN) guidelines for cervical cancer were changed to make OH the gold standard treatment for patients with early-stage cervical cancer.

Controversial but important issues remain on the potential peritoneal contamination of tumor cells during colpotomy at the end of a radical hysterectomy. The use of a uterine manipulator and/or not closing the vaginal cuff before colpotomy may facilitate tumor spread in the peritoneal cavity and can lead to poor oncological outcomes [6]. The LACC trial did not address these specific issues. In the LACC trial, 84.4% of the MIH group were conventional laparoscopy, and only 15.6% were Robot-Assisted Surgery (RAS) [4]. In the retrospective study of Cusimano et al., which confirmed the results of the LACC trial, the distribution of laparoscopic and robotic surgery was 89.6% and 10.4%, respectively [7]. However, in many countries, this does not reflect the actual allocation of conventional laparoscopy vs RAS [8,9]. As a laparoscopic radical hysterectomy is seen as one of the most difficult gynecological cancer surgeries with a long learning curve, the oncological outcomes of a robotic-assisted approach, with a shorter learning curve, have not been fully investigated yet. 

Given the potential of peritoneal spread of tumor cells during the colpotomy phase of an MIH, large retrospective cohort studies have been published on the survival of MIH with vaginal cuff closure as a way to limit tumor cell spillage [6,8,10-12]. With this approach, the disease-free and overall survival seems to be similar to those of the OH group in the LACC trial [6,10]. The first modalities described for vaginal cuff closure were a vaginal approach during a radical vaginal hysterectomy (Shauta operation) and a radical vaginal trachelectomy (Dargent’s operation) [12]. In 2019, Kanao et al. introduced the “no-look, no-touch technique” for laparoscopic radical hysterectomy [13]. The current ongoing Robotic Approach for Cervical Cancer (RACC) trial uses a plastic cable tie, which is placed at the end of the robotic-assisted radical hysterectomy around the vaginal cuff and tightened to close the vagina [8].

The LACC trial was a large international and multicenter study with nine-year patient recruitment. However, it may raise the question of adequate operator proficiency for robotic surgery in each center, as only a small group of 50 RAS patients was included in this trial. More recently, using RAS in specialized centers has shown comparable oncological outcomes to OH [14,15]. Here we describe with a video the different key steps during a robotic radical hysterectomy for vaginal cuff preparation and, for the first time, the application of a barbed suture at the end of the robotic radical hysterectomy to close the vaginal cuff with a purse string suture.

This technique was presented as part of a lecture given during the oncology session at the Annual Meeting of the European Society of Gynaecological Endoscopy (ESGE) in Brussels on the 4th of October 2023.

## Technical report

A 42-year-old Caucasian G2 P2 A0 with a new diagnosis of early-stage cervical cancer was referred to our tertiary cancer center. She presented with symptoms of irregular vaginal bleeding. Her last PAP smear was dated from more than 10 years ago. She had no other relevant medical history or family history. During the first visit to our hospital, a gynecological examination showed an irregular cervical lesion, predominantly on the right side of the cervix. There was no apparent extension towards the vaginal walls. The lesion showed irregular vascularity. An echogenic vascular zone of 18x7mm, color score 3, was seen on a transvaginal ultrasound scan without evidence of invasion in the paracervical tissues or the sacro-uterine ligaments. Colposcopy-directed biopsies were taken in the referring hospital, and pathology revealed a well-differentiated squamous cell carcinoma. Imaging with Magnetic Resonance Imaging (MRI) described a tumor in the uterine cervix, mainly on the right side, invading the cervical stroma for approximately 50%. There was no evidence of invasion elsewhere. An abdomen/chest CT showed no suspicious lymph nodes or metastasis. She was staged, according to the International Federation of Gynecology and Obstetrics (FIGO), as a FIGO-stage 1B1, as the tumor was less than 2cm on imaging. The patient was discussed at the Multidisciplinary Oncology Consultation, and a robotic radical hysterectomy with vaginal cuff closure, with bilateral pelvic sentinel lymph node dissection, was proposed after informed consent of the patient. The vaginal cuff was closed, before colpotomy, with a circular purse string barbed suture. Final histology revealed a poorly differentiated squamous cell carcinoma with a superficial width of 24.4mm and a depth of invasion of 7.9mm into the cervical stroma. No lymphovascular invasion was observed. The bilateral pelvic sentinel lymph nodes were negative on histology. There were no peri- or postoperative complications. She was discharged after 48 hours with a bladder catheter, which was removed after seven days. During follow-up, the patient did not experience any sexual or urinary discomfort or pelvic pain symptoms.

Robotic surgical technique

**Figure 1:**
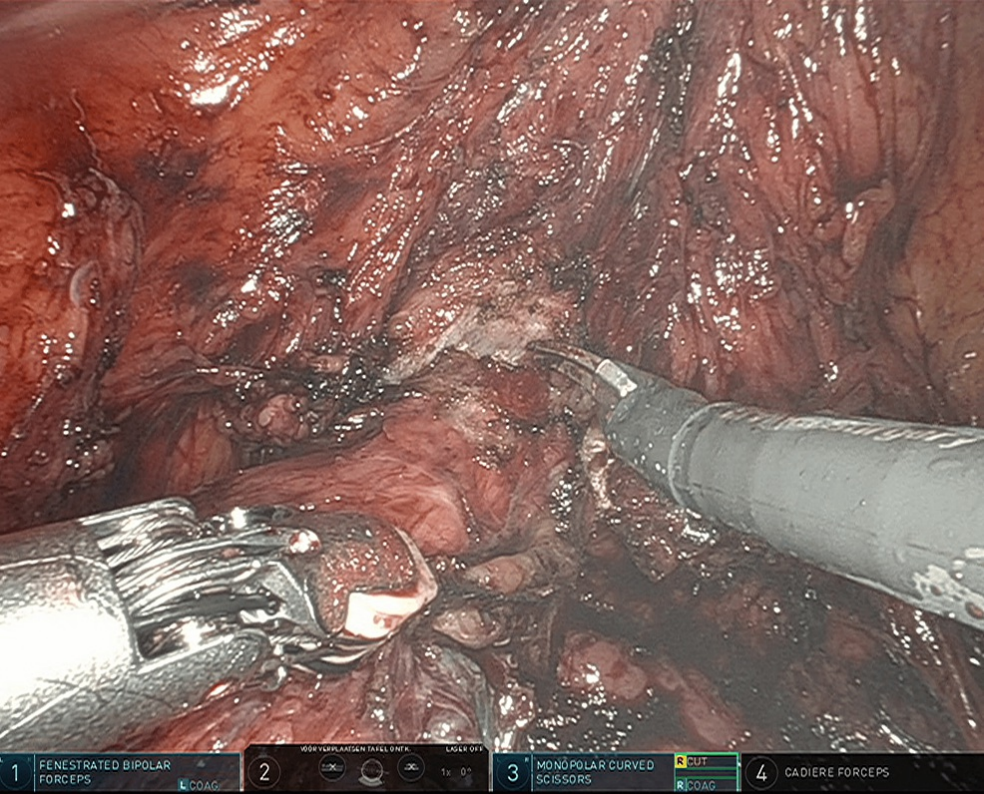
Dissection and incision of the vesicovaginal fascia with unipolar scissor.

**Figure 2:**
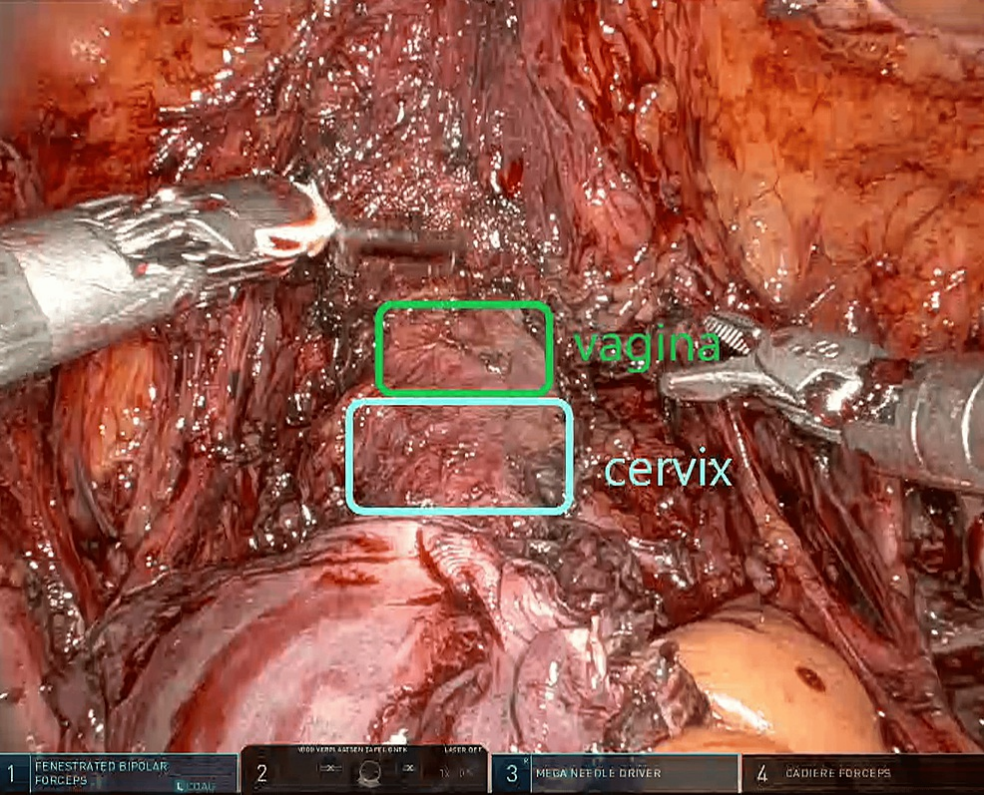
Vaginal cuff of approximately 2-3cm.

The video demonstrates the key surgical steps of a robotic-assisted radical hysterectomy, Querleu-Morrow type B, prior to the vaginal cuff preparation and closure. No uterine manipulator was used. The broad ligament was opened with subsequent lateralization of the (meso-)ureter, and the Okabayashi pararectal space was identified with the inferior hypogastric nerve. Subsequently, the ureter was tunneled with dissection and transection of the paracervical tissues at the level of and medially of the ureter. To prepare the anterior and posterior part of the vaginal cuff, the vesicovaginal and rectovaginal septum was dissected with transection of the bladder pillars anteriorly and the uterosacral ligaments posteriorly. Finally, the vesicovaginal fascia was dissected with the unipolar scissor (Video 1; Figures 1, 2).

**Video 1:** Robotic vaginal cuff closure during radical hysterectomy for early-stage cervical cancer: the Bruges method.

**Figure 3:**
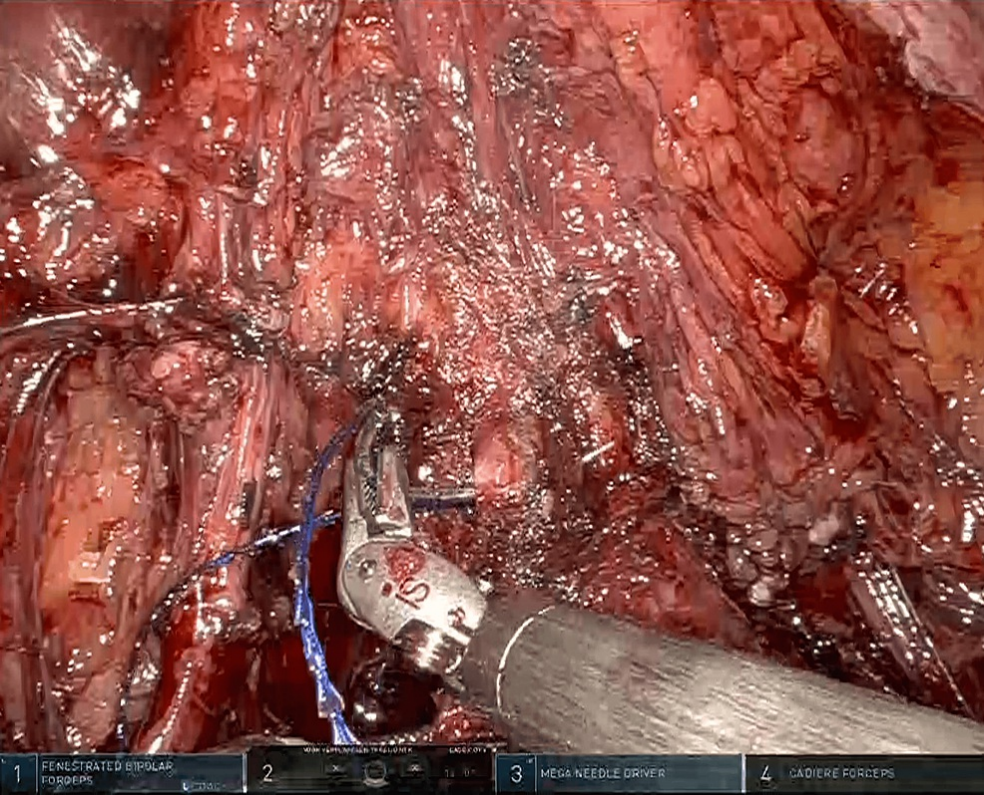
With a barbed suture, StratafixTM 0, going through the anterior/posterior and lateral vaginal walls to create a circular purse string suture to close the vaginal cuff.

**Figure 4:**
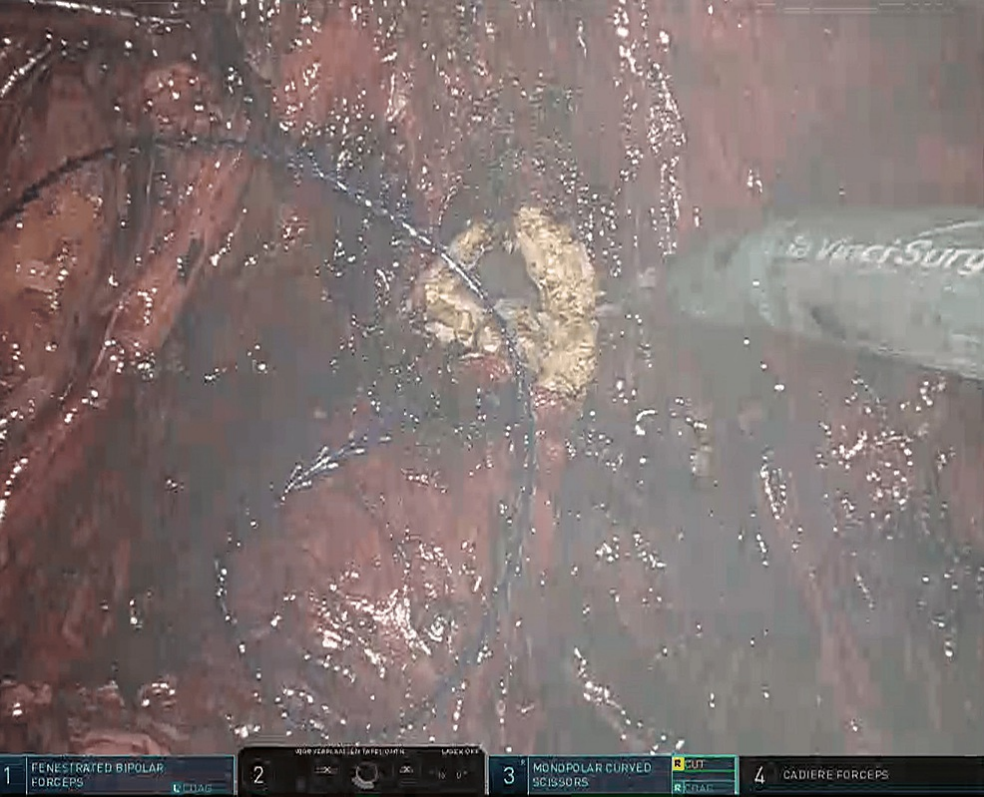
With unipolar scissor opening the vagina distally from the closed vaginal cuff.

The vaginal cuff was closed intracorporeally during the robotic procedure. A knotless, absorbable, barbed suture (StratafixTM 0, alternatively V-LocTM 0) was inserted via the assistant trocar. A purse string suture was placed circumferentially in the vaginal walls, where the vesicovaginal fascia was cut with diathermy, at approximately 2-3cm distance of the exocervix, under vaginal guidance: the barbed suture going twice through the anterior vaginal wall, once through the right lateral wall, twice through the posterior vaginal wall, once through the left lateral wall, and ending through the anterior vaginal wall twice for tightening the circular barbed suture, to close the vagina (Video 1, Figures 3, 4). 

The suture thread was kept long. After the vaginal cuff closure, the colpotomy was performed using the monopolar scissor, just distal to the purse string suture (Figure 4). The uterus was removed transvaginally by the assistant, using the long end of the suture thread as guidance toward the resection specimen. The remaining open vaginal top was closed with separate Vicryl 1 stitches.

## Discussion

In 2018, the Laparoscopic Approach for Cervical Cancer (LACC) trial demonstrated higher incidences of recurrence and death in patients treated by minimally invasive radical hysterectomy (MIH) compared to open radical hysterectomy (OH) for early-stage cervical cancer. The trial suggested a 3.74-fold higher rate of recurrence and a 6.00-fold higher rate of all-cause death in patients treated by MIH compared to OH [4]. Subsequent large retrospective studies confirmed these findings: Cusimano et al. showed a recurrence of 16.2% in MIH compared to 8.4% in OH in patients with FIGO-stage IB cervical cancer. The five-year cumulative incidence of death in this group was 12.5% in MIH compared to 5.4% in OH [7]. Melamed et al. reported a statistically significant difference in risk of death within 4 years: 9.1% in the MIH group compared to 5.3% in the OH group [5].

The SUCCOR (Surgery in Cervical Cancer, Observational, Retrospective) study, a large retrospective cohort study in Europe [6], is of special interest. This study confirmed the outcomes of the LACC trial, namely that MIH has worse oncologic outcomes than OH. However, the study showed that two important interventions in the MIH group could lead to similar oncologic outcomes than the OH group, in particular, restraining the use of a uterine manipulator and/or making a protective vaginal cuff closure. The hypothesis is that with this approach, there is minimal to no spillage of tumor cells into the peritoneal cavity. Disease-free survival at 4.5 years was 74% for MIH without vaginal cuff closure compared to 93% for MIH with vaginal cuff closure [6].

The predomination of conventional laparoscopy compared to robotic-assisted surgery (RAS) in the trials mentioned above has to be taken into account [4,6,7]. Two large nationwide cohort studies suggested that RAS in centralized, specialized centers does not seem to give worse outcomes than open surgery [14,15]. In Sweden, all cervical cancer care is centralized in 7 university hospitals, with only a few gynecological surgeons per center performing RAS. The study of Alfonzo et al. showed similar results for both OH and RAS: a 5-year overall survival rate of 92% and 94%, respectively, and a disease-free survival rate of 84% and 88%, respectively [14].

Similarly, since Denmark had a gradual but nearly complete national adoption of RAS for early-stage cervical cancer, they could compare the prognoses before and after the introduction of RAS [15]. The 5-year overall survival was 92.3% in the group before RAS introduction and 94.4% after RAS introduction [15]. No difference was observed in the 5-year disease-free survival: 91.8% vs 91% in the respective groups [15]. The ongoing RACC (Robot-assisted Approach to Cervical Cancer) trial will further investigate whether RAS is a safe alternative to laparotomy in early-stage cervical cancer [8].

The Bruges method, described here, is a new alternative for protective vaginal cuff closure using a circular purse string barbed suture, before colpotomy, during a robotic radical hysterectomy without using a uterine manipulator. Multiple studies have suggested the oncologic benefit of vaginal cuff closure before colpotomy [10,16,17]. It has been proven that a protective vaginal cuff before MIH leads to no tumoral spillage intraperitoneally [17]. According to Kohler et al., a 4.5-year disease-free survival rate after a combined vaginal/laparoscopic approach is 95.8% in early-stage cervical cancer [10]. Important in their technique is the creation of a protective vaginal cuff and the avoidance of the use of a uterine manipulator [10]. These results are nearly identical to those of the open radical hysterectomy group in the LACC trial. A systematic review and meta-analysis on the survival of laparo-assisted vaginal radical hysterectomy with a protective vaginal cuff showed comparable survival rates as the OH-group in the LACC trial [16].

In 1994, Fanning et al. described and compared absorbable staples, for transection of the vaginal cuff, with classic suture ligation in Rutledge class III open abdominal radical hysterectomy and demonstrated similar oncologic outcomes for both techniques [18]. Since the 1990s, little has been reported on preventative/protective measures to close the vaginal cuff to avoid tumor spillage into the peritoneal cavity during an open abdominal radical hysterectomy for early-stage cervical cancer. Daniel Dargent and Denis Querleu, both French gynecological cancer surgeons, pioneered in the 1990s the vaginal approach of protective vaginal cuff closure during radical vaginal hysterectomy (or trachelectomy) or during laparoscopic-assisted radical vaginal hysterectomy [12].

Currently, most authors developed methods to make a protective vaginal cuff closure vaginally by using Chrobak forceps or several sutures after a circular incision of the vaginal mucosa [10,12]. These techniques are a possible addendum to all different approaches for radical hysterectomy (open, laparoscopic, or robotic-assisted). A possible disadvantage is the long learning curve of these often difficult vaginal approaches [16]. For a minimally invasive approach (laparoscopic or robotic), these additional vaginal surgical techniques could imply a longer operation time, compared to our robotic technique, because of the initial need to perform vaginal surgery. Our robotic approach for vaginal cuff closure gives the advantage of direct visualization of the bladder, ureters, and rectum intra-abdominally while making the circular purse string suture around the vaginal cuff, which could lead to a potentially lower rate of urinary dysfunction or rectum injuries.

Some intracorporeal approaches during MIH have been described by Falconer et al., Boyraz et al., and Mun et al. [8,19,20]. Falconer et al., in the RACC trial, use a plastic cable tie which is placed, at the end of the robotic-assisted radical hysterectomy, around the vaginal cuff and tightened to close the vagina [8]. This is an easy, quick, and low-cost surgical approach. However, as this technique brings a cable tie around the exterior vaginal walls, not through the vaginal walls, it is uncertain what the potential risk is of the cable tie slipping off the vaginal walls. Boyraz et al. and Mun et al. used staplers during laparoscopic radical hysterectomy [19,20]. Advantages of this technique are the easiness of the method and the tightly closed vaginal cuff. A possible disadvantage of this technique is the straight cutting of a circular object (the vagina), causing the lateral sides of the resected vaginal wall to be longer than the medial part. The Bruges method, described here, could signify a more equal resection of the upper part of the vagina after robotically closing the vaginal cuff. Being able to choose the location of the vaginal cuff precisely is particularly important since a vaginal cuff length of 2cm or less is associated with more local recurrences and lower survival rates.

Studies like the SOLUTION (Safety Of Laparoscopic or Robotic Radical Surgery Using an Endoscopic sTapler for Inhibiting tumOr spillage of cervical Neoplasms) trial and the RACC trial will contribute to a better knowledge on the oncologic safety of minimally invasive surgery in early-stage cervical cancer [8,11].

## Conclusions

Important safety measures during radical hysterectomy for early-stage cervical cancer are to avoid tumor spillage into the peritoneal cavity by safely removing the lymph nodes, avoiding the use of a uterine manipulator, and by closing the vaginal cuff before colpotomy. We present here a new technique of vaginal cuff closure, in a robotic intracorporeal way, during robotic-assisted radical hysterectomy, with the application of a purse string barbed suture. Our technique is a feasible, relatively fast, and easy-to-learn addition to robotic radical hysterectomy in early-stage cervical cancer. Since it can be done during the robotic procedure, the technique has the general advantages of minimally invasive surgery.
